# Estimation of finite population distribution function with dual use of auxiliary information under non-response

**DOI:** 10.1371/journal.pone.0243584

**Published:** 2020-12-17

**Authors:** Sardar Hussain, Sohaib Ahmad, Sohail Akhtar, Amara Javed, Uzma Yasmeen

**Affiliations:** 1 Department of Statistics, Quaid-i-Azam University, Islamabad, Pakistan; 2 Department of Statistics, Government College University, Lahore, Pakistan; 3 School of Statistics, Minhaj University, Lahore, Pakistan; 4 Institute of Molecular Biology and Biotechnology, The University of Lahore, Lahore, Pakistan; Tongii University, CHINA

## Abstract

In this paper, we propose two new families of estimators for estimating the finite population distribution function in the presence of non-response under simple random sampling. The proposed estimators require information on the sample distribution functions of the study and auxiliary variables, and additional information on either sample mean or ranks of the auxiliary variable. We considered two situations of non-response (i) non-response on both study and auxiliary variables, (ii) non-response occurs only on the study variable. The performance of the proposed estimators are compared with the existing estimators available in the literature, both theoretically and numerically. It is also observed that proposed estimators are more precise than the adapted distribution function estimators in terms of the percentage relative efficiency.

## 1. Introduction

One of the common problems in sample surveys is non-response. The non-response bias is serious concern in survey studies. It occurs in many ways, including linguistic problems, illness, due to response, due to non-acceptance, the process of return address misguided, captured by another person, etc. Sometime, sample survey experts use the auxiliary information to improve the precision of estimators. As expected, non-response not only reduces the precision of an estimator but also increases its bias. A number of research articles have been published on the estimation of population mean under non-response in order to control the non-response bias and to increase the efficiency of estimators. Hansen and Hurwitz [1] suggested that a sub-sample of earlier non-respondents can be re-communicated with a more expensive system. They adapted the first effort by a mail questionnaire and the second attempt by personal interview. In addition, firstly, they developed an estimator to estimate the population mean in the presence of non-response. For some related works on the non-response, we refer to Rao [2], Khare and Srivastava [3], Khare and Sinha [4, 5], Olufadi and Kumar [6], Muneer et al. [7], Pal and Singh [8], Ahmad and Shabbir [9] and the references cited therein.

The problem of estimating the finite population cumulative distribution function (CDF) arises when the interest lies in knowing the proportion of values of the study variable that are less or equal to a certain value. There are different situations where estimating the CDF is deemed necessary. For example, for an economist, it is interesting to know the proportion of the population that 27% or more Pakistanis do not have skills. Similarly, a soil scientist may be interested in estimating the distribution of clay percent in the soil. In addition, policy-makers may be interested in knowing the proportion of people living in a developing country below the poverty line.

In survey sampling literature, the authors have estimated the CDF using information on one or more auxiliary variables. Chambers and Dunstan [10] suggested an estimator for estimating the CDF that requires information both on the study and auxiliary variables. On similar lines, Rao et al. [11] and Rao [12] proposed ratio and difference/regression estimators for estimating the CDF under a general sampling design. Kuk [13] suggested a kernel method for estimating the CDF using the auxiliary information. Ahmed Abu-Dayyeh [14] estimated the CDF using information on multiple auxiliary variables. Chen and Wu [15] suggested a method for estimating the CDF and quantiles using the model-calibrated pseudo empirical likelihood. A calibration approach has been used by Rueda et al. [16] to devise an estimator for estimating the CDF. Singh et al. [17] considered the problem of estimating the CDF and quantiles with the use of auxiliary information at the estimation stage of a survey. Moreover, Chen et al. [18] investigate the injury severities of truck drivers in single-and multi-vehicle accidents on rural highways, Zeng et al. [19] worked on a multivariate random-parameters tobit model for analyzing highway crash rates by injury severity, Yaqub and Shabbir [20] considered a generalized class of estimators for estimating the CDF in the presence of non-response. Dong et al. [21] investigating the differences of single-vehicle and multi-vehicle accident probability using mixed logit model, Chen et al. [22] worked on analysis of hourly crash likelihood using unbalanced panel data mixed logit model and real-time driving environmental big data, Zeng et al. [23] suggested a jointly modeling area-level crash rates by severity and Zeng et al. [24] used spatial joint analysis for zonal daytime and night time crash frequencies using a bayesian bivariate conditional autoregressive model. Hussain et al. [25] proposed two new families of estimators using the supplementary information on auxiliary variable and exponential function for the population distribution functions in case of non-response under simple random sampling, and Hussain et al. [26] two new families of estimators for estimating the finite population distribution function are proposed under simple and stratified random sampling schemes using supplementary information on the distribution function, mean and ranks of the auxiliary variable.

In this paper, we propose two new families of estimators for estimating the CDF using information on the sample distribution function, mean and ranks of the auxiliary variable along with the information on the sample distribution function of the study variable under simple random sampling in the presence of non-response. The bias and mean squared errors (MSEs) of the existing and proposed estimators of the CDF are derived under the first order of approximation. The theoretical and numerical comparisons revealed that the proposed estimators are more precise than the existing adapted estimators when estimating the CDF.

The issue of non-response in survey sampling threatens to undermine the validity of inferences drawn from estimates based on those surveys. High non-response rates create opportunity or risk for bias in estimates and affect survey design, data collection, estimation and analysis Plewes et al. [27]. With these issues in mind, we propose two new families of estimators for estimating the CDF using information on the sample distribution function, mean and ranks of the auxiliary variable along with the information on the sample distribution function of the study variable under simple random sampling in the presence of non-response. The bias and mean squared errors (MSEs) of the existing and proposed estimators of the CDF are derived under the first order of approximation. The theoretical and numerical comparisons revealed that the proposed estimators are more precise than the existing adapted estimators when estimating the CDF.

The rest of the paper is organized as follows. In Section 2, some notations are given. In Section 3, we adapt some estimators of the finite population mean for estimating the finite CDF. The proposed estimators are given in Section 4. In Sections 5 and 6, theoretical and numerical comparisons are conducted, respectively. Finally, conclusions are drawn in Section 7.

## 2. Notations

Consider a finite population Ω = {*U*_1_, *U*_2_,…,*U*_*N*_} of *N* distinct units, which is partitioned into *N*_1_ respondents $\Omega_{1}=\{U_{1},U_{2},\ldots ,U_{N_{1}}\}$ and *N*_2_ non-respondents $\Omega_{2}=\{U_{1},U_{2},\ldots ,U_{N_{2}}\}$, where *N* = *N*_1_+*N*_2_. A sample of size *n* units is drawn from this population using simple random sampling without replacement. It is assumed that out of *n* units, *n*_1_ units respond but *n*_2_ = *n*−*n*_1_ units do not respond. Clearly, *n*_1_ and *n*_2_ units belong to the respondent and non-respondent groups, respectively. Moreover, a sub- sample of size *r* = *n*_2_/*k* units, where *k*>1, is drawn from *n*_2_ units using simple random sampling without replacement, and, this time the response is obtained from *r* units. Let

*Y*: the study variable,
*X*: the auxiliary variable,
*Z*: ranks of *X*,
*I*(*Y*≤*y*): indicator variable based on *Y*,
*I*(*X*≤*x*): indicator variable based on *X*,
$F(y)=\sum_{i=1}^{N}‍I(Y_{i}\leq y)/N$: the population distribution function of *I*(*Y*≤*y*),
$\hat{F}(y)=\sum_{i=1}^{n}‍I(Y_{i}\leq y)/n$: the sample distribution function of *I*(*Y*≤*y*),
$F(x)=\sum_{i=1}^{N}‍I(X\leq x)/N$: the population distribution function of *I*(*X*≤*x*),
$\hat{F}(x)=\sum_{i=1}^{n}‍I(X_{i}\leq x)/n$: the sample distribution function of *I*(*X*≤*x*),
$\bar{X}=\sum_{i=1}^{N}‍X_{i}/N$: the population mean of *X*,
$\hat{\bar{X}}=\sum_{i=1}^{n}‍X_{i}/n$: the sample mean of *X*,
$\bar{Z}=\sum_{i=1}^{N}‍Z_{i}/N$: the population mean of *Z*,
$\hat{\bar{Z}}=\sum_{i=1}^{n}‍Z_{i}/n$: the sample mean of *Z*,
$F_{\left(2\right)}(y)=\sum_{i=1}^{N_{2}}‍I(Y_{i}\leq y)/N_{2}$: the population distribution function of *I*(*Y*≤*y*) for non-response group,
$F_{\left(2\right)}(x)=\sum_{i=1}^{N_{2}}‍I(X_{i}\leq x)/N_{2}$: the population distribution function of *I*(*X*≤*x*) for non-response group,
${\bar{X}}_{\left(2\right)}=\sum_{i=1}^{N_{2}}‍X_{i}/N_{2}$: the population mean of *X* for non-response group,
${\bar{Z}}_{\left(2\right)}=\sum_{i=1}^{N_{2}}‍Z_{i}/N_{2}$: the population mean of *Z* for non-response group,
${\hat{F}}_{\left(1\right)}(y)=\sum_{i=1}^{n_{1}}‍I(Y_{i}\leq y)/n_{1}$ denote the sample distribution function based on *n*_1_ responding units out of *n* units,
${\hat{F}}_{\left(1\right)}(x)=\sum_{i=1}^{n_{1}}‍I(X_{i}\leq x)/n_{1}$ denote the sample distribution function based on *n*_1_ responding units out of *n* units,
${\bar{X}}_{\left(1\right)}=\sum_{i=1}^{n_{1}}‍X_{i}/n_{1}$ denote the sample mean based on *n*_1_ responding units out of *n* units,
${\bar{Z}}_{\left(1\right)}=\sum_{i=1}^{n_{1}}‍Z_{i}/n_{1}$ denote the sample mean based on *n*_1_ responding units out of *n* units,
${\hat{F}}_{\left(2r\right)}(y)=\sum_{i=1}^{r}‍I(Y_{i}\leq y)/r$ denote the sample distribution function based on *r* responding units out of *n*_2_ non-response units,
${\hat{F}}_{\left(2r\right)}(x)=\sum_{i=1}^{r}‍I(X_{i}\leq x)/r$ denote the sample distribution function based on *r* responding units out of *n*_2_ non-response units,
${\bar{X}}_{\left(2r\right)}=\sum_{i=1}^{r}‍X_{i}/r$ denote the sample mean based on *r* responding units out of *n*_2_ non-response units,
${\bar{Z}}_{\left(2r\right)}=\sum_{i=1}^{r}‍Z_{i}/r$ denote the sample mean based on *r* responding units out of *n*_2_ non-response units,
$S_{1}^{2}=\sum_{i=1}^{N}‍{\left(I(Y_{i}\leq y)-F(y)\right)}^{2}/(N-1)$: the population variance of *I*(*Y*≤*y*),
$S_{2}^{2}=\sum_{i=1}^{N}‍{\left(I(X_{i}\leq x)-F(x)\right)}^{2}/(N-1)$: the population variance of *I*(*X*≤*x*),
$S_{3}^{2}=\sum_{i=1}^{N}‍(X_{i}-\bar{X}{)}^{2}/(N-1)$: the population variance of *X*,
$S_{4}^{2}=\sum_{i=1}^{N}‍(Z_{i}-\bar{Z}{)}^{2}/(N-1)$: the population variance of *Z*,
$S_{1(2)}^{2}=\sum_{i=1}^{N_{2}}‍{\left(I(Y_{i}\leq y)-F_{\left(2\right)}(y)\right)}^{2}/(N_{2}-1)$: the population variance of *I*(*Y*≤*y*) for non-response group,
$S_{2(2)}^{2}=\sum_{i=1}^{N_{2}}‍{\left(I(X_{i}\leq x)-F_{\left(2\right)}(x)\right)}^{2}/(N_{2}-1)$: the population variance of *I*(*X*≤*x*) for non-response group,
$S_{3(2)}^{2}=\sum_{i=1}^{N_{2}}‍(X_{i}-{\bar{X}}_{\left(2\right)}{)}^{2}/(N_{2}-1)$: the population variance of *X* for non-response group,
$S_{4(2)}^{2}=\sum_{i=1}^{N_{2}}‍(Z_{i}-{\bar{Z}}_{\left(2\right)}{)}^{2}/(N_{2}-1)$: the population variance of *Z* for non-response group,
*C*_1_ = *S*_1_/*F*(*y*): the population coefficient of variation of *I*(*Y*≤*y*),
*C*_2_ = *S*_2_/*F*(*x*): the population coefficient of variation of *I*(*X*≤*x*),
$C_{3}=S_{3}/\bar{X}$: the population coefficient of variation of *X*,
$C_{4}=S_{4}/\bar{Z}$: the population coefficient of variation of *Z*,
*C*_1(2)_ = *S*_1(2)_/*F*_(2)_(*y*): the population coefficient of variation of *I*(*Y*≤*y*) for non-response group,
*C*_2(2)_ = *S*_2(2)_/*F*_(2)_(*x*): the population coefficient of variation of *I*(*X*≤*x*) for non-response group,
$C_{3(2)}=S_{3(2)}/{\bar{X}}_{\left(2\right)}$: the population coefficient of variation of *X* for non-response group,
$C_{4(2)}=S_{4(2)}/{\bar{Z}}_{\left(2\right)}$: the population coefficient of variation of *Z* for non-response group,
$S_{12}=\sum_{i=1}^{N}‍\{\left(I(Y_{i}\leq y)-F(y))(I(X_{i}\leq x)-F(x)\right)\}/(N-1)$: the population covariance
between *I*(*Y*≤*y*) and *I*(*X*≤*x*),
$S_{13}=\sum_{i=1}^{N}‍\{\left(I(Y_{i}\leq y)-F(y))(X_{i}-\bar{X}\right)\}/(N-1)$: the population covariance
between *I*(*Y*_*i*_≤*y*) and *X*,
$S_{23}=\sum_{i=1}^{N}‍\{\left(I(X_{i}\leq x)-F(x))(X_{i}-\bar{X}\right)\}/(N-1)$: the population covariance
between *I*(*X*≤*x*) and *X*,
$S_{14}=\sum_{i=1}^{N}‍\{\left(I(Y_{i}\leq y)-F(y))(Z_{i}-\bar{Z}\right)\}/(N-1)$: the population covariance
between *I*(*Y*≤*y*) and *Z*,
$S_{24}=\sum_{i=1}^{N}‍\{\left(I(X_{i}\leq x)-F(x))(Z_{i}-\bar{Z}\right)\}/(N-1)$: the population covariance
between *I*(*X*≤*x*) and *Z*,
$S_{12(2)}=\sum_{i=1}^{N_{2}}‍\{\left(I(Y_{i}\leq y)-F_{\left(2\right)}(y))(I(X_{i}\leq x)-F_{\left(2\right)}(x)\right)\}/(N_{2}-1)$: the population covariance between *I*(*Y*≤*y*) and *I*(*X*≤*x*) for non-response group,
$S_{13(2)}=\sum_{i=1}^{N_{2}}‍\{\left(I(Y_{i}\leq y)-F_{\left(2\right)}(y))(X_{i}-{\bar{X}}_{\left(2\right)}\right)\}/(N_{2}-1)$: the population covariance
between *I*(*Y*≤*y*) and *X* for non-response group,
$S_{23(2)}=\sum_{i=1}^{N_{2}}‍\{\left(I(X_{i}\leq x)-F_{\left(2\right)}(x))(X_{i}-{\bar{X}}_{\left(2\right)}\right)\}/(N_{2}-1)$: the population covariance
between *I*(*X*≤*x*) and *X* for non-response group,
$S_{14(2)}=\sum_{i=1}^{N_{2}}‍\{\left(I(Y_{i}\leq y)-F_{\left(2\right)}(y))(Z_{i}-{\bar{Z}}_{\left(2\right)}\right)\}/(N_{2}-1)$: the population covariance
between *I*(*Y*≤*y*) and *Z* for non-response group,
$S_{24(2)}=\sum_{i=1}^{N_{2}}‍\{\left(I(X_{i}\leq x)-F_{2}(x))(Z_{i}-{\bar{Z}}_{2}\right)\}/(N_{2}-1)$: the population covariance
between *I*(*X*≤*x*) and *Z* for non-response group,
Δ_12_ = *S*_12_/(*S*_1_*S*_2_): the population correlation coefficient between *I*(*Y*≤*y*) and *I*(*X*≤*x*),
Δ_13_ = *S*_13_/(*S*_1_*S*_3_): the population correlation coefficient between *I*(*Y*≤*y*) and *X*,
Δ_23_ = *S*_23_/(*S*_2_*S*_3_): the population correlation coefficient between *I*(*X*≤*x*) and *X*,
Δ_14_ = *S*_14_/(*S*_1_*S*_4_): the population correlation coefficient between *I*(*Y*≤*y*) and *Z*,
Δ_24_ = *S*_24_/(*S*_2_*S*_4_): the population correlation coefficient between *I*(*X*≤*x*) and *Z*,
∇_12(2)_ = *S*_12(2)_/(*S*_1(2)_*S*_2(2)_): the population correlation coefficient between *I*(*Y*≤*y*) and *I*(*X*≤*x*) for non-response group,
∇_13(2)_ = *S*_13(2)_/(*S*_1(2)_*S*_3(2)_): the population correlation coefficient between *I*(*Y*≤*y*) and *X* for non-response group,
∇_23(2)_ = *S*_23(2)_/(*S*_2(2)_*S*_3(2)_): the population correlation coefficient between *I*(*X*≤*x*) and *X* for non-response group,
∇_14(2)_ = *S*_14(2)_/(*S*_1(2)_*S*_4(2)_): the population correlation coefficient between *I*(*Y*≤*y*) and *Z* for non-response group,
∇_24(2)_ = *S*_24(2)_/(*S*_2(2)_*S*_4(2)_): the population correlation coefficient between *I*(*X*≤*x*) and *Z* for non-response group.

We can write

$$F(y)=W_{1}F_{\left(1\right)}(y)+W_{2}F_{\left(2\right)}(y)$$ (1)

Similarly, let

$$F(x)=W_{1}F_{\left(1\right)}(x)+W_{2}F_{\left(2\right)}(x)$$ (2)

Let

$$\bar{X}=W_{1}{\bar{X}}_{\left(1\right)}+W_{2}{\bar{X}}_{\left(2\right)}$$ (3)

Similarly let

$$\bar{Z}=W_{1}{\bar{Z}}_{\left(1\right)}+W_{2}{\bar{Z}}_{\left(2\right)}$$ (4)

where *W*_*j*_ = *N*_*j*_/*N*, $F_{j}(y)=\sum_{i=1}^{N_{j}}‍I(Y_{i}\leq y)/N_{j}$, for *j* = 1,2., $F_{j}(x)=\sum_{i=1}^{N_{j}}‍I(X_{i}\leq x)/N_{j}$, ${\bar{X}}_{j}=\sum_{i=1}^{N_{j}}‍X_{i}/N_{j}$ and ${\bar{Z}}_{j}=\sum_{i=1}^{N_{j}}‍Z_{i}/N_{j}$.

Following Hansen and Hurwitz [1], Yaqub and Shabbir [20] have suggested an unbiased estimator of *F*(*y*) under non-response, which is given by

$${\hat{F}}^{*}(y)=w_{1}{\hat{F}}_{\left(1\right)}(y)+w_{2}{\hat{F}}_{\left(2r\right)}(y)$$

with its variance

$$Var({\hat{F}}^{*}(y))=\lambda S_{1}^{2}+\lambda_{2}S_{1(2)}^{2},$$ (5)

where *w*_*j*_ = *n*_*j*_/*n* for *j* = 1,2, *λ* = (1/*n*−1/*N*) and *λ*_2_ = *W*_2_(*k*−1).

Similarly, let

$$1{\hat{F}}^{*}(x)=w_{1}{\hat{F}}_{\left(1\right)}(x)+w_{2}{\hat{F}}_{\left(2r\right)}(x),$$

$${\hat{\bar{X}}}^{*}=w_{1}{\bar{X}}_{\left(1\right)}+w_{2}{\bar{X}}_{\left(2r\right)}\;and$$

$${\hat{\bar{Z}}}^{*}=w_{1}{\bar{Z}}_{\left(1\right)}+w_{2}{\bar{Z}}_{\left(2r\right)},$$

are unbiased estimators of *F*(*x*), $\bar{X}$ and $\bar{Z}$, respectively, under non-response, with corresponding variances

$$1Var({\hat{F}}^{*}(x))=\lambda S_{1}^{2}+\lambda_{2}S_{1(2)}^{2},$$

$$Var({\hat{\bar{X}}}^{*})=\lambda S_{2}^{2}+\lambda_{2}S_{2(2)}^{2}\;and$$

$$Var({\hat{\bar{Z}}}^{*})=\lambda S_{3}^{2}+\lambda_{2}S_{3(2)}^{2},$$

respectively.

In order to obtain the biases and MSEs of the existing and proposed estimators, the following relative error terms are considered. Let

$$e_{1}^{*}=\frac{{\hat{F}}^{*}(y)-F(y)}{F(y)},e_{2}^{*}=\frac{{\hat{F}}^{*}(x)-F(x)}{F(x)},e_{3}^{*}=\frac{{\hat{\bar{X}}}^{*}-\bar{X}}{\bar{X}},e_{4}^{*}=\frac{{\hat{\bar{Z}}}^{*}-\bar{Z}}{\bar{Z}},$$

$$e_{2}=\frac{\hat{F}(x)-F(x)}{F(x)},e_{3}=\frac{\hat{\bar{X}}-\bar{X}}{\bar{X}},e_{4}=\frac{\hat{\bar{Z}}-\bar{Z}}{\bar{Z}},$$

such that $E(e_{i}^{*})=E(e_{i})=0$ for i* = 1,2,3,4 and *i* = 2,3,4, where *E*(⋅) stands for the mathematical expectation of (⋅). Let

$$V_{rstu}=E[e_{1}^{r}\;e_{2}^{s}\;e_{3}^{t}\;e_{4}^{u}]\;and\;V_{rstu}^{*}=E[e_{1}^{*r}\;e_{2}^{*s}\;e_{3}^{*t}\;e_{4}^{*u}],$$

where *r*,*s*,*t*,*u* = 1,2,3,4, Here,

$$E(e_{1}^{*2})=\lambda C_{1}^{2}+\lambda_{2}C_{1(2)}^{2}=V_{2000}^{*2},E(e_{2}^{*2})=\lambda C_{2}^{2}+\lambda_{2}C_{2(2)}^{2}=V_{0200}^{*2},$$

$$E(e_{3}^{*2})=\lambda C_{3}^{2}+\lambda_{2}C_{3(2)}^{2}=V_{0020}^{*2},E(e_{4}^{*2})=\lambda C_{4}^{2}+\lambda_{2}C_{4(2)}^{2}=V_{0002}^{*2},$$

$$E(e_{2}^{2})=\lambda C_{2}^{2}=V_{0200},E(e_{3}^{2})=\lambda C_{3}^{2}=V_{0020},E(e_{4}^{2})=\lambda C_{4}^{2}=V_{0002}^{2},$$

$$E(e_{1}^{*}e_{2}^{*})=\lambda \Delta_{12}C_{1}C_{2}+\lambda_{2}\nabla_{12(2)}C_{1(2)}C_{2(2)}=V_{1100}^{*},$$

$$E(e_{1}^{*}e_{3}^{*})=\lambda \Delta_{13}C_{1}C_{3}+\lambda_{2}\nabla_{13(2)}C_{1(2)}C_{3(2)}=V_{1010}^{*},$$

$$E(e_{1}^{*}e_{4}^{*})=\lambda \Delta_{14}C_{1}C_{4}+\lambda_{2}\nabla_{14(2)}C_{1(2)}C_{4(2)}=V_{1001}^{*},$$

$$E(e_{2}^{*}e_{3}^{*})=\lambda \Delta_{23}C_{2}C_{3}+\lambda_{2}\nabla_{23(2)}C_{2(2)}C_{3(2)}=V_{0110}^{*},$$

$$E(e_{2}^{*}e_{4}^{*})=\lambda \Delta_{24}C_{2}C_{4}+\lambda_{2}\nabla_{24(2)}C_{2(2)}C_{4(2)}=V_{0101}^{*},$$

$$E(e_{1}^{*}e_{2})=\lambda \Delta_{12}C_{1}C_{2}=V_{1100},E(e_{1}^{*}e_{3})=\lambda \Delta_{13}C_{1}C_{3}=V_{1010},$$

$$E(e_{1}^{*}e_{4})=\lambda \Delta_{14}C_{1}C_{4}=V_{1001},\;E(e_{2}e_{3})=\lambda \Delta_{23}C_{2}C_{3}=V_{0110},$$

$$E(e_{2}e_{4})=\lambda \Delta_{24}C_{2}C_{4}=V_{0101}.$$

Let $\Delta_{1.23}^{2}=\left(\frac{V_{1100}^{*2}V_{0020}^{*}+V_{1010}^{*2}V_{0200}^{*}-2V_{1010}^{*}V_{1100}^{*}V_{0110}^{*}}{V_{2000}^{*}(V_{0200}^{*}V_{0020}^{*}-V_{0110}^{*2})}\right)$: the coefficient of multiple

determination of *I*(*Y*≤*y*) on *I*(*X*≤*x*) and *X* with situation-I under non-response.

Let $\nabla_{1.23(2)}^{2}=\left(\frac{V_{1100}^{2}V_{0020}+V_{1010}^{2}V_{0200}-2V_{1010}V_{1100}V_{0110}}{V_{2000}^{*}(V_{0200}V_{0020}-V_{0110}^{2})}\right)$: the coefficient of multiple

determination of *I*(*Y*≤*y*) on *I*(*X*≤*x*) and *X* with situation-II under non-response.

Let $\Delta_{1.24}^{2}=\left(\frac{V_{1100}^{*2}V_{0002}^{*}+V_{1001}^{*2}V_{0200}^{*}-2V_{1001}^{*}V_{1100}^{*}V_{0101}^{*}}{V_{2000}^{*}(V_{0200}^{*}V_{0002}^{*}-V_{0101}^{*2})}\right)$: the coefficient of multiple

determination of *I*(*Y*≤*y*) on *I*(*X*≤*x*) and *Z* with situation-I under non-response.

Let $\nabla_{1.24(2)}^{2}=\left(\frac{V_{1100}^{2}V_{0002}+V_{1001}^{2}V_{0200}-2V_{1001}V_{1100}V_{0101}}{V_{2000}^{*}(V_{0200}V_{0002}-V_{0101}^{2})}\right)$: the coefficient of multiple

determination of *I*(*Y*≤*y*) on *I*(*X*≤*x*) and *Z* with situation-II under non-response.

Under non-response, two situations are considered. The situation-I refers to the non-response both on study and auxiliary variables while situation-II refers to the non-response only on the study variable. For notational convenience, we follow the notations given in Table 1.

**Table 1:** Estimators, variances, covariances and correlation under non-response situations.

Situation		I	II
Estimator			
${\hat{F}}_{H}(y)$	=	${\hat{F}}^{*}(y)$	${\hat{F}}^{*}(y)$
${\hat{F}}_{H}(x)$	=	${\hat{F}}^{*}(x)$	$\hat{F}(x)$
${\hat{\bar{X}}}_{H}$	=	${\hat{\bar{X}}}^{*}$	$\hat{\bar{X}}$
${\hat{\bar{Z}}}_{H}$	=	${\hat{\bar{Z}}}^{*}$	$\hat{\bar{Z}}$
	Variance/covariance		
*Q*_*rstu*_	=	$V_{rstu}^{*}$	*V*_*rstu*_
*Q*_2000_	=	$V_{2000}^{*}$	*V*_2000_
*Q*_0200_	=	$V_{0200}^{*}$	*V*_0200_
*Q*_0020_	=	$V_{0020}^{*}$	*V*_0020_
*Q*_0002_	=	$V_{0002}^{*}$	*V*_0002_
*Q*_1100_	=	$V_{1100}^{*}$	*V*_1100_
*Q*_1010_	=	$V_{1010}^{*}$	*V*_1010_
*Q*_1001_	=	$V_{1001}^{*}$	*V*_1001_
*Q*_0110_	=	$V_{0110}^{*}$	*V*_0110_
*Q*_0101_	=	$V_{0101}^{*}$	*V*_0101_
Coefficient of correlation			
*ϱ*_12_	=	Δ_12_	∇_12(2)_
*ϱ*_13_	=	Δ_13_	∇_13(2)_
*ϱ*_23_	=	Δ_23_	∇_23(2)_
*ϱ*_14_	=	Δ_14_	∇_14(2)_
*ϱ*_24_	=	Δ_24_	∇_24(2)_
Coefficient of multiple determination			
$\varrho_{1.23}^{2}$	=	$\Delta_{1.23}^{2}$	$\nabla_{1.23}^{2}$
$\varrho_{1.24}^{2}$	=	$\Delta_{1.24}^{2}$	$\nabla_{1.24}^{2}$

## 3. Adapted estimators

In this section, some estimators of finite population mean are adapted for estimating the finite CDF under non-response with simple random sampling. Moreover, the biases and MSEs of these adapted estimators are derived under the first order of approximation.

1. Cochran [28] adapted ratio estimator of *F*(*y*) is
  $${\hat{F}}_{2}(y)={\hat{F}}_{H}(y)(\frac{F(x)}{{\hat{F}}_{H}(x)}).$$ (6)
  The bias and MSE of ${\hat{F}}_{2}(y)$, to the first order of approximation, are
  $$Bias({\hat{F}}_{2}(y))≅F(y)(Q_{0200}-Q_{1100}),$$
  $$MSE({\hat{F}}_{2}(y))≅F^{2}(y)(Q_{2000}+Q_{0200}-2Q_{1100}).$$ (7)
2. Murthy [29] adapted product estimator of *F*(*y*) is
  $${\hat{F}}_{3}(y)={\hat{F}}_{H}(y)\left(\frac{{\hat{F}}_{H}(x)}{F(x)}\right).$$ (8)
  The bias and MSE of ${\hat{F}}_{3}(y)$, to the first order of approximation, are
  $$Bias({\hat{F}}_{3}(y))=F(y)Q_{1100},$$
  $$MSE({\hat{F}}_{3}(y))≅F^{2}(y)(Q_{2000}+Q_{0200}+2Q_{1100}).$$ (9)
3. The adapted difference estimator of *F*(*y*) is
  $${\hat{F}}_{4}(y)={\hat{F}}_{H}(y)+k(F(x)-{\hat{F}}_{H}(x)),$$ (10)
  where *k* is an unknown constant. Here, ${\hat{F}}_{4}(y)$ is an unbiased estimator of $\hat{F}(y)$. The minimum variance of ${\hat{F}}_{4}(y)$ at the optimum value *k*_(opt)_ = (*F*(*y*)*Q*_1100_)/(*F*(*x*)*Q*_0200_) is
  $$Var_{min}({\hat{F}}_{4}(y))=\frac{F^{2}(y)(Q_{2000}Q_{0200}-Q_{1100}^{2})}{Q_{0200}}.$$ (11)
  Here, (11) may be written as
  $$Var_{min}({\hat{F}}_{4}(y))=F^{2}(y)Q_{2000}(1-\varrho_{12}^{2}).$$ (12)
4. Rao [30] adapted difference-type estimator of *F*(*y*) is
  $${\hat{F}}_{5}(y)=k_{1}{\hat{F}}_{H}(y)+k_{2}(F(x)-{\hat{F}}_{H}(x)),$$ (13)
  where *k*_1_ and *k*_2_ are unknown constants. The bias and MSE of ${\hat{F}}_{5}(y)$, to the first order of approximation, are
  $$Bias({\hat{F}}_{5}(y))=F(y)(k_{1}-1),$$
  $$MSE({\hat{F}}_{5}(y))≅F^{2}(y)-2k_{1}F^{2}(y)+k_{1}^{2}F^{2}(y)+k_{1}^{2}F^{2}(y)Q_{2000}$$
  $$-2k_{1}k_{2}F(y)F(x)Q_{1100}+k_{2}^{2}F^{2}(x)Q_{0200}.$$ (14)
  The optimum values of *k*_1_ and *k*_2_, determined by minimizing (??), are
  $$k_{1(opt)}=\frac{Q_{0200}}{\left(Q_{0200}Q_{2000}-Q_{1100}^{2}+Q_{0200}\right)},$$
  $$k_{2(opt)}=\frac{F(y)Q_{1100}}{F(x)(Q_{2000}Q_{0200}-Q_{1100}^{2}+Q_{0200})}.$$
  The minimum MSE of ${\hat{F}}_{5}(y)$ at the optimum values of *k*_1_ and *k*_2_ is
  $$MSE_{min}({\hat{F}}_{5}(y))=\frac{F^{2}(y)(Q_{2000}Q_{0200}-Q_{1100}^{2})}{\left(Q_{2000}Q_{0200}-Q_{1100}^{2}+Q_{0200}\right)}.$$ (15)
  Here, (15) may be written as
  $$MSE_{min}({\hat{F}}_{5}(y))=\frac{F^{2}(y)Q_{2000}(1-\varrho_{12}^{2})}{1+Q_{2000}(1-\varrho_{12}^{2})}.$$ (16)
5. Singh et al. [31] adapted generalized ratio-type exponential estimator of *F*(*y*) is
  $${\hat{F}}_{6}(y)={\hat{F}}_{H}(y)exp\left(\frac{a(F(x)-{\hat{F}}_{H}(x))}{a(F(x)+{\hat{F}}_{H}(x))+2b}\right),$$ (17)
  where *a* and *b* are known constants. The bias and MSE of ${\hat{F}}_{6}(y)$, to the first order of approximation, are
  $$Bias({\hat{F}}_{6}(y))≅F(y)\left(\frac{3}{8}\theta^{2}Q_{0200}-\frac{1}{2}\theta Q_{1100}\right),$$
  $$MSE({\hat{F}}_{6}(y))≅\frac{F^{2}(y)}{4}(4Q_{2000}+\theta^{2}Q_{0200}-4\theta Q_{1100}),$$ (18)
  where *θ* = *aF*(*x*)/(*aF*(*x*)+*b*).
6. Grover and Kaur [32] adapted generalized class of ratio-type exponential estimator of *F*(*y*) is
  $${\hat{F}}_{7}(y)=\{k_{3}{\hat{F}}_{H}(y)+k_{4}(F(x)-{\hat{F}}_{H}(x))\}exp\left(\frac{a(F(x)-{\hat{F}}_{H}(x))}{a(F(x)+{\hat{F}}_{H}(x))+2b}\right),$$ (19)
  where *k*_3_ and *k*_4_ are unknown constants. The bias and MSE of ${\hat{F}}_{7}(y)$, to the first order of approximation, are
  $$Bias({\hat{F}}_{7}(y))≅F(y)(k_{3}-1)+\frac{3}{8}\theta^{2}k_{3}F(y)+\frac{1}{2}\theta k_{4}F(x)Q_{0200}-\frac{1}{2}\theta F(y)Q_{1100},$$
  $$MSE({\hat{F}}_{7}(y))≅k_{4}^{2}F^{2}(x)Q_{0200}+k_{3}^{2}F^{2}(y)Q_{2000}+2\theta k_{3}k_{4}F(y)F(x)Q_{0200}$$
  $$-2k_{3}k_{4}F(y)F(x)Q_{1100}+F^{2}(y)-2k_{3}F^{2}(y)+\theta k_{3}^{2}F^{2}(y)$$
  $$+k_{3}F^{2}(y)Q_{1100}-\theta k_{4}F(y)F(x)Q_{0200}-2\theta k_{3}^{2}F^{2}(y)Q_{1100}$$
  $$-\frac{3}{4}\theta^{2}k_{3}F^{2}(y)Q_{0200}+\theta^{2}k_{3}^{2}F^{2}(y)Q_{0200}.$$ (20)
  The optimum values of *k*_3_ and *k*_4_, determined by minimizing (??), are
  $$k_{3(opt)}=\frac{Q_{0200}(\theta^{2}Q_{0200}-8)}{8(-Q_{2000}Q_{0200}+Q_{1100}^{2}-Q_{0200})},$$
  $$k_{4(opt)}=\frac{F(y)(\theta^{3}Q_{0200}^{2}-\theta^{2}Q_{0200}Q_{1100}+4\theta Q_{2000}Q_{0200}-4\theta Q_{1100}^{2}-4\theta Q_{0200}+8Q_{1100})}{8F(x)(Q_{2000}Q_{0200}-Q_{1100}^{2}+Q_{0200})}.$$
  The simplified minimum MSE of ${\hat{F}}_{7}(y)$ at the optimum values of *k*_3_ and *k*_4_ is
  $$MSE_{min}({\hat{F}}_{7}(y))≅\frac{F^{2}(y)}{64}\left(64-16\theta^{2}Q_{0200}-\frac{Q_{0200}(-8+\theta^{2}Q_{0200}{)}^{2}}{Q_{0200}(1+Q_{2000})-Q_{1100}^{2}}\right).$$ (21)
  Here, (21) may be written as
  $$MSE_{min}({\hat{F}}_{7}(y))≅Var_{min}({\hat{F}}_{4}(y))-\frac{F^{2}(y){\left(\theta^{2}Q_{0200}^{2}-8Q_{1100}^{2}+8Q_{0200}Q_{2000}\right)}^{2}}{64Q_{0200}^{2}\{1+Q_{2000}(1-\varrho_{12}^{2})\}},$$ (22)
  which shows that ${\hat{F}}_{7}(y)$ is more precise than ${\hat{F}}_{4}(y)$.

## 4. Proposed estimators

The precision of an estimator surges by using the appropriate secondary information at the estimation stage. In previous studies, the sample distribution function of the auxiliary variable was used to expand the productivities of the prevailing distribution function estimators. In a recent study, Haq et al. [33] recommended that to use ranks of the auxiliary variable as an additional auxiliary variable to increase the precision of an estimator of the population mean. On the similar lines use additional auxiliary information on sample means of the auxiliary and ranked-auxiliary variables along with the sample distribution function estimators of *F*(*y*) and *F*(*x*) to estimate the finite CDF. For this persists, we recommend two families of estimators for estimating *F*(*y*). The (first and second) families of estimators, used the (sample mean of the auxiliary variable and the sample mean of the ranked-auxiliary variable) as an additional auxiliary variable.

On the lines of ${\hat{F}}_{5}(y)$ and ${\hat{F}}_{6}(y)$, first proposed family of estimators for estimating *F*(*y*) is given by

$${\hat{F}}_{8}(y)=\left\{k_{5}{\hat{F}}_{H}(y)+k_{6}(\frac{F(x)-{\hat{F}}_{H}(x)}{F(x)})(\frac{\bar{X}-{\hat{\bar{X}}}_{H}}{\bar{X}})\right\}exp\left(\frac{a(F(x)-{\hat{F}}_{H}(x))}{a(F(x)+{\hat{F}}_{H}(x))+2b}\right),$$ (23)

where *k*_5_, *k*_6_ and *k*_7_ are unknown constants, *a* (≠0) and *b* are either two real numbers or functions of known population parameters of *I*(*X*≤*x*), like *R*_12_, *β*_2_ (coefficient of kurtosis), *C*_2_, etc.

The estimator ${\hat{F}}_{8}(y)$ can also be written as

$${\hat{F}}_{8}(y)=\{k_{5}F(y)(1+e_{1})-k_{6}e_{2}-k_{7}e_{3}\}\left(1-\frac{1}{2}\theta e_{2}+\frac{3}{8}\theta^{2}e_{2}^{2}+\cdots \right).$$ (24)

Simplifying (24) and keeping terms only up to the second power of *e*_*i*_’s, we can write

$$({\hat{F}}_{8}(y)-F(y))=-F(y)+k_{5}F(y)+k_{5}F(y)e_{1}-\frac{1}{2}\theta k_{5}F(y)e_{2}-k_{6}e_{2}-k_{7}e_{3}$$

$$+\frac{3}{8}\theta^{2}k_{5}F(y)e_{2}^{2}+\frac{1}{2}\theta k_{6}e_{2}^{2}-\frac{1}{2}\theta k_{5}F(y)e_{1}e_{2}+\frac{1}{2}\theta k_{7}e_{2}e_{3}.$$ (25)

The bias and MSE of ${\hat{F}}_{8}(y)$, to the first order of approximation, are

$$Bias({\hat{F}}_{8}(y))≅F(y)(k_{5}-1)+\frac{3}{8}\theta^{2}k_{5}F(y)Q_{0200}+\frac{1}{2}\theta k_{6}Q_{0200}-\frac{1}{2}\theta k_{5}F(y)Q_{1100}+\frac{1}{2}\theta k_{7}Q_{0110},$$

$$MSE({\hat{F}}_{8}(y))≅F^{2}(y)(k_{5}-1{)}^{2}+k_{5}^{2}F^{2}(y)Q_{2000}+k_{6}^{2}Q_{0200}+k_{7}^{2}Q_{0020}+\theta^{2}k_{5}^{2}F^{2}(y)Q_{0200}$$

$$-\theta k_{6}F(y)Q_{0200}+2\theta k_{5}k_{6}F(y)Q_{0200}-\frac{3}{4}\theta^{2}k_{5}F^{2}(y)Q_{0200}+\theta k_{5}F^{2}(y)Q_{1100}$$

$$-2\theta k_{5}^{2}F^{2}(y)Q_{1100}-2k_{5}k_{6}F(y)Q_{1100}-2k_{5}k_{7}F(y)Q_{1010}-\theta k_{7}F(y)Q_{0110}$$

$$+2\theta k_{5}k_{7}F(y)Q_{0110}-2k_{6}k_{7}Q_{0110}.$$ (26)

The optimum values of *k*_5_, *k*_6_ and *k*_7_, determined by minimizing (26), are

$$k_{5(opt)}=\frac{8-\theta^{2}Q_{0200}}{8\{1+Q_{2000}(1-\varrho_{1.23}^{2})\}},$$

$$k_{6(opt)}=\frac{F(y)[\begin{array}{l} \;\theta^{3}Q_{0200}^{3/2}(\varrho_{23}^{2}-1)+Q_{2000}^{1/2}(-8+\theta^{2}Q_{0200})(\varrho_{12}-\varrho_{23}\varrho_{13})\\ \;+4\theta Q_{0200}^{1/2}(\varrho_{23}^{2}-1)\{-1+Q_{2000}(1-\varrho_{1.23}^{2})\} \end{array}]}{8Q_{0200}^{1/2}(\varrho_{23}^{2}-1)\{-1+Q_{2000}(1-\varrho_{1.23}^{2})\}},$$

$$k_{7(opt)}=\frac{F(y)Q_{2000}^{1/2}(8-\theta^{2}Q_{0200})(\varrho_{12}-\varrho_{23}\varrho_{13})}{8Q_{0200}^{1/2}(\varrho_{23}^{2}-1)\{-1+Q_{2000}(1-\varrho_{1.23}^{2})\}}.$$

The simplified minimum MSE of ${\hat{F}}_{8}(y)$, at the optimum values of *k*_5_, *k*_6_ and *k*_7_ is

$$MSE_{min}({\hat{F}}_{8}(y))≅\frac{F^{2}(y)\{64Q_{2000}(1-\varrho_{1.23}^{2})-\theta^{4}Q_{0200}^{2}-16\theta^{2}Q_{0200}Q_{2000}(1-\varrho_{1.23}^{2})\}}{64\{1+Q_{2000}(1-\varrho_{1.23}^{2})\}},$$ (27)

where $\varrho_{1.23}^{2}=\left(\frac{Q_{1100}^{2}Q_{0020}+Q_{1010}^{2}Q_{0200}-2Q_{1010}Q_{1100}Q_{0110}}{Q_{2000}(Q_{0200}Q_{0020}-Q_{0110}^{2})}\right)$.

Here, (27) may be written as

$$MSE_{min}({\hat{F}}_{8}(y))≅Var_{min}({\hat{F}}_{4}(y))-H_{1}-H_{2},$$ (28)

where

$$H_{1}=\frac{F^{2}(y){\left(\theta^{2}Q_{0200}^{2}-8Q_{1100}^{2}+8Q_{0200}Q_{2000}\right)}^{2}}{64Q_{0200}^{2}\{1+Q_{2000}(1-\varrho_{12}^{2})\}}\;and$$

$$H_{2}=\frac{F^{2}(y){\left(\theta^{2}Q_{0200}-8\right)}^{2}{\left(Q_{0200}Q_{1010}-Q_{0110}Q_{1100}\right)}^{2}}{64Q_{0200}^{2}Q_{0020}(1-\varrho_{23}^{2})\{1+Q_{2000}(1-\varrho_{12}^{2})\}\{1+Q_{2000}(1-\varrho_{1.23}^{2})\}}.$$

It is clear that ${\hat{F}}_{8}(y)$ is more precise than ${\hat{F}}_{4}(y)$.

On similar lines, second proposed family of estimators for estimating *F*(*y*) is given by

$${\hat{F}}_{9}(y)=\left\{k_{8}\hat{F}(y)+k_{9}(\frac{F(x)-\hat{F}(x)}{F(x)})+k_{10}(\frac{\bar{Z}-\hat{\bar{Z}}}{\bar{Z}})\right\}exp\left(\frac{a(F(x)-\hat{F}(x))}{a(F(x)-\hat{F}(x))+2b}\right),$$ (29)

where *k*_8_, *k*_9_ and *k*_10_ are unknown constants, *a*(≠0) and *b* are either two real numbers or functions of known population parameters of *I*(*X*≤*x*), like *R*_12_, *β*_2_ (coefficient of kurtosis), *C*_2_, etc. The estimator ${\hat{F}}_{9}(y)$ can also be written as

$${\hat{F}}_{9}(y)=\{k_{8}F(y)(1+e_{1})-k_{9}e_{2}-k_{10}e_{4}\}\left(1-\frac{1}{2}\theta e_{2}+\frac{3}{8}\theta^{2}e_{2}^{2}+\cdots \right).$$ (30)

Simplifying (30) and keeping terms only up to the second power of *e*_*i*_’s, we can write

$$({\hat{F}}_{9}(y)-F(y))=-F(y)+k_{8}F(y)+k_{8}F(y)e_{1}-\frac{1}{2}\theta k_{8}F(y)e_{2}-k_{9}e_{2}-k_{10}e_{4}$$

$$+\frac{3}{8}\theta^{2}k_{8}F(y)e_{2}^{2}+\frac{1}{2}\theta k_{9}e_{2}^{2}-\frac{1}{2}\theta k_{8}F(y)e_{1}e_{2}+\frac{1}{2}\theta k_{10}e_{2}e_{4}.$$ (31)

The bias and MSE of ${\hat{F}}_{9}(y)$, to the first order of approximation, are

$$Bias({\hat{F}}_{9}(y))≅F(y)(k_{8}-1)+\frac{3}{8}\theta^{2}k_{8}F(y)Q_{0200}+\frac{1}{2}\theta k_{9}Q_{0200}-\frac{1}{2}\theta k_{8}F(y)Q_{1100}+\frac{1}{2}\theta k_{9}Q_{0101},$$

$$MSE({\hat{F}}_{9}(y))≅F^{2}(y)(k_{8}-1{)}^{2}+k_{8}^{2}F^{2}(y)Q_{2000}+k_{9}^{2}Q_{0200}+k_{10}^{2}Q_{0002}+\theta^{2}k_{8}^{2}F^{2}(y)Q_{0200}$$

$$-\theta k_{9}F(y)Q_{0200}+2\theta k_{8}k_{9}F(y)Q_{0200}-\frac{3}{4}\theta^{2}k_{8}F^{2}(y)Q_{0200}+\theta k_{8}F^{2}(y)Q_{1100}$$

$$-2\theta k_{8}^{2}F^{2}(y)Q_{1100}-2k_{8}k_{9}F(y)Q_{1100}-2k_{8}k_{10}F(y)Q_{1001}-\theta k_{10}F(y)Q_{0101}$$

$$+2\theta k_{8}k_{10}F(y)Q_{0101}-2k_{9}k_{10}Q_{0101}.$$ (32)

The optimum values of *k*_8_, *k*_9_ and *k*_10_, determined by minimizing (??), are

$$k_{8(opt)}=\frac{8-\theta^{2}Q_{0200}}{8\{1+Q_{2000}(1-\varrho_{1.24}^{2})\}},$$

$$k_{9(opt)}=\frac{F(y)[\begin{array}{l} \;\theta^{3}Q_{0200}^{3/2}(\varrho_{24}^{2}-1)+Q_{2000}^{1/2}(-8+\theta^{2}Q_{0200})(\varrho_{12}-R_{24}R_{14})\\ \;+4\theta Q_{0200}^{1/2}(\varrho_{24}^{2}-1)\{-1+Q_{2000}(1-\varrho_{1.24}^{2})\} \end{array}]}{8Q_{0200}^{1/2}(\varrho_{24}^{2}-1)\{-1+Q_{2000}(1-\varrho_{1.24}^{2})\}},$$

$$k_{10(opt)}=\frac{F(y)Q_{2000}^{1/2}(8-\theta^{2}Q_{0200})(\varrho_{12}-\varrho_{24}\varrho_{14})}{8Q_{0200}^{1/2}(\varrho_{24}^{2}-1)\{-1+Q_{2000}(1-\varrho_{1.24}^{2})\}}.$$

The simplified minimum MSE of ${\hat{F}}_{9}(y)$ at the optimum values of *k*_8_, *k*_9_ and *k*_10_ is

$$MSE_{min}({\hat{F}}_{9}(y))≅\frac{F^{2}(y)\{64Q_{2000}(1-\varrho_{1.24}^{2})-\theta^{4}Q_{0200}^{2}-16\theta Q_{0200}Q_{2000}(1-\varrho_{1.24}^{2})\}}{64\{1+Q_{2000}(1-\varrho_{1.24}^{2})\}},$$ (33)

where $\varrho_{1.24}^{2}=\left(\frac{Q_{1100}^{2}Q_{0002}+Q_{1001}^{2}Q_{0200}-2Q_{1001}Q_{1100}Q_{0101}}{Q_{2000}(Q_{0200}Q_{0002}-Q_{0101}^{2})}\right)$.

Here, (33) may be written as

$$MSE_{min}({\hat{F}}_{9}(y))≅Var_{min}({\hat{F}}_{4}(y))-H_{1}-H_{3},$$ (34)

where

$$H_{1}=\frac{F^{2}(y){\left(\theta^{2}Q_{0200}^{2}-8Q_{1100}^{2}+8Q_{0200}\right)}^{2}}{64Q_{0200}^{2}\{1+Q_{2000}(1-R_{12}^{2})\}}\;and$$

$$H_{3}=\frac{F^{2}(y){\left(\theta^{2}Q_{0200}-8\right)}^{2}{\left(Q_{0200}Q_{1001}-Q_{0101}Q_{1100}\right)}^{2}}{64Q_{0200}^{2}Q_{0002}(1-R_{24}^{2})\{1+Q_{2000}(1-R_{12}^{2})\}\{1+Q_{2000}(1-R_{1.24}^{2})\}}.$$

It is clear that ${\hat{F}}_{9}(y)$ is more precise than ${\hat{F}}_{4}(y)$.

In Table 2, we put some members of the Singh et al. [31], Grover and Kaur [32], and proposed families of estimators with selected choices of *a* and *b*.

**Table 2:** Some members of the adapted and proposed distribution function estimators.

*a*	*b*	${\hat{F}}_{6}(y)$	${\hat{F}}_{7}(y)$	${\hat{F}}_{8}(y)$	${\hat{F}}_{9}(y)$
1	*C*_2_	${\hat{F}}_{6}^{\left(1\right)}(y)$	${\hat{F}}_{7}^{\left(1\right)}(y)$	${\hat{F}}_{8}^{\left(1\right)}(y)$	${\hat{F}}_{9}^{\left(1\right)}(y)$
1	*β*_2_	${\hat{F}}_{6}^{\left(2\right)}(y)$	${\hat{F}}_{7}^{\left(2\right)}(y)$	${\hat{F}}_{8}^{\left(2\right)}(y)$	${\hat{F}}_{9}^{\left(2\right)}(y)$
*β*_2_	*C*_2_	${\hat{F}}_{6}^{\left(3\right)}(y)$	${\hat{F}}_{7}^{\left(3\right)}(y)$	${\hat{F}}_{8}^{\left(3\right)}(y)$	${\hat{F}}_{9}^{\left(3\right)}(y)$
*C*_2_	*β*_2_	${\hat{F}}_{6}^{\left(4\right)}(y)$	${\hat{F}}_{7}^{\left(4\right)}(y)$	${\hat{F}}_{8}^{\left(4\right)}(y)$	${\hat{F}}_{9}^{\left(4\right)}(y)$
1	12	${\hat{F}}_{6}^{\left(5\right)}(y)$	${\hat{F}}_{7}^{\left(5\right)}(y)$	${\hat{F}}_{8}^{\left(5\right)}(y)$	${\hat{F}}_{9}^{\left(5\right)}(y)$
*C*_2_	*R*_12_	${\hat{F}}_{6}^{\left(6\right)}(y)$	${\hat{F}}_{7}^{\left(6\right)}(y)$	${\hat{F}}_{8}^{\left(6\right)}(y)$	${\hat{F}}_{9}^{\left(6\right)}(y)$
*R*_12_	*C*_2_	${\hat{F}}_{6}^{\left(7\right)}(y)$	${\hat{F}}_{7}^{\left(7\right)}(y)$	${\hat{F}}_{8}^{\left(7\right)}(y)$	${\hat{F}}_{9}^{\left(7\right)}(y)$
*β*_2_	*R*_12_	${\hat{F}}_{6}^{\left(8\right)}(y)$	${\hat{F}}_{7}^{\left(8\right)}(y)$	${\hat{F}}_{8}^{\left(8\right)}(y)$	${\hat{F}}_{9}^{\left(8\right)}(y)$
*R*_12_	*β*_2_	${\hat{F}}_{6}^{\left(9\right)}(y)$	${\hat{F}}_{7}^{\left(9\right)}(y)$	${\hat{F}}_{8}^{\left(9\right)}(y)$	${\hat{F}}_{9}^{\left(9\right)}(y)$
1	*NF*(*x*)	${\hat{F}}_{6}^{\left(10\right)}(y)$	${\hat{F}}_{7}^{\left(10\right)}(y)$	${\hat{F}}_{8}^{\left(1\right)}(y)$	${\hat{F}}_{9}^{\left(10\right)}(y)$

## 5. Efficiency comparisons in simple random sampling

In this section, the adapted and proposed estimators of *F*(*y*) are compared in terms of the minimum MSEs. [(i)]

1. From (5) and (28),
  $$MSE_{min}({\hat{F}}_{8}(y))<Var({\hat{F}}_{1}(y))if$$
  $$F^{2}(y)Q_{2000}\varrho_{12}^{2}+H_{1}+H_{2}>0.$$
2. From (7) and (28),
  $$MSE_{min}({\hat{F}}_{8}(y))<MSE({\hat{F}}_{2}(y))if$$
  $$\frac{F^{2}(y)}{Q_{2000}}{\left(Q_{0200}-Q_{1100}\right)}^{2}+H_{1}+H_{2}>0.$$
3. From (9) and (28),
  $$MSE_{min}({\hat{F}}_{8}(y))<MSE({\hat{F}}_{3}(y))if$$
  $$\frac{F^{2}(y)}{Q_{2000}}{\left(Q_{0200}+Q_{1100}\right)}^{2}+H_{1}+H_{2}>0.$$
4. From (12) and (28),
  $$MSE_{min}({\hat{F}}_{8}(y))<MSE_{min}({\hat{F}}_{4}(y))if$$
  $$H_{1}+H_{2}>0.$$
5. From (16) and (28),
  $$MSE_{min}({\hat{F}}_{8}(y))<MSE_{min}({\hat{F}}_{5}(y))if$$
  $$\frac{F^{2}(y)\theta^{2}Q_{0200}\{\theta^{2}Q_{0200}+16Q_{2000}(1-\varrho_{12}^{2})\}}{64\{1+Q_{2000}(1-\varrho_{12}^{2})\}}+H_{2}>0.$$
6. From (18) and (28),
  $$MSE_{min}({\hat{F}}_{8}(y))<MSE({\hat{F}}_{6}(y))if$$
  $$\frac{F^{2}(y)}{Q_{2000}}{\left(\frac{\theta Q_{0200}}{2}-Q_{1100}\right)}^{2}+H_{1}+H_{2}>0.$$
7. From (22) and (28),
  $$MSE_{min}({\hat{F}}_{8}(y))<MSE_{min}({\hat{F}}_{7}(y))if$$
  $$H_{2}>0.$$
8. From (5) and (34),
  $$MSE_{min}({\hat{F}}_{9}(y))<Var({\hat{F}}_{1}(y))if$$
  $$\frac{1}{Q_{0200}}F^{2}(y)Q_{1100}^{2}+H_{1}+H_{3}>0.$$
9. From (7) and (34),
  $$MSE_{min}({\hat{F}}_{9}(y))<MSE({\hat{F}}_{2}(y))if$$
  $$\frac{F^{2}(y)}{Q_{2000}}{\left(Q_{0200}-Q_{1100}\right)}^{2}+H_{1}+H_{3}>0.$$
10. From (9) and (34),
  $$MSE_{min}({\hat{F}}_{9}(y))<MSE({\hat{F}}_{3}(y))if$$
  $$\frac{F^{2}(y)}{Q_{2000}}{\left(Q_{0200}+Q_{1100}\right)}^{2}+H_{1}+H_{3}>0.$$
11. From (12) and (34),
  $$MSE_{min}({\hat{F}}_{9}(y))<MSE_{min}({\hat{F}}_{4}(y))if$$
  $$H_{1}+H_{3}>0.$$
12. From (16) and (34),
  $$MSE_{min}({\hat{F}}_{9}(y))<MSE_{min}({\hat{F}}_{5}(y))if$$
  $$\frac{F^{2}(y)\theta^{2}Q_{0200}\{\theta^{2}Q_{0200}+16Q_{2000}(1-\varrho_{12}^{2})\}}{64\{1+Q_{2000}(1-R_{12}^{2})\}}+H_{3}>0.$$
13. From (18) and (34),
  $$MSE_{min}({\hat{F}}_{9}(y))<MSE_{min}({\hat{F}}_{6}(y))if$$
  $$\frac{F^{2}(y)}{Q_{2000}}{\left(\frac{\theta Q_{0200}}{2}-Q_{1100}\right)}^{2}+H_{1}+H_{3}>0.$$
14. From (22) and (34),
  $$MSE_{min}({\hat{F}}_{9}(y))<MSE_{min}({\hat{F}}_{7}(y))if$$
  $$H_{3}>0.$$
  The proposed families of estimators are always more precise than the adapted estimators as the above conditions (i)–(xiv) are always true.

## 6. Empirical study

In this section, we conduct a numerical study to see the performance of the adapted and proposed distribution function estimators. For this purpose, five population are considered. The summary statistics of these populations are reported in Tables 3–6. The percentage relative efficiency (PRE) of an estimator ${\hat{F}}_{i}(y)$ with respect to ${\hat{F}}_{H}(y)$ is

$$PRE({\hat{F}}_{i}(y),{\hat{F}}_{H}(y))=\frac{Var({\hat{F}}_{H}(y))}{MSE_{min}({\hat{F}}_{i}(y))}\times 100,$$

where *i* = 2,3,…,9.

**Table 3:** Summary statistics for population I.

Parameter	Value	Parameter	Value
*N*	50	*S*_2_	0.50508
*n*	15	*S*_3_	21805
*λ*	0.04667	*S*_4_	15756
$\bar{X}$	75.8720	*R*_12_	−0.20000
$\bar{R}$	25.5000	*R*_13_	0.25176
*F*(*y*)	0.50000	*R*_23_	−0.79517
*F*(*x*)	0.50000	*R*_14_	0.21345
*S*_1_	0.50508	*R*_24_	−0.86628
Non-response			
Parameter	Value	Parameter	Value
*N*_2_	12	*S*_4(2)_	3.60555
*w*_2_	0.24000	*R*_12(2)_	−0.50709
*λ*_2_	0.01600	*R*_13(2)_	0.35848
*S*_1(2)_	0.51493	*R*_23(2)_	−0.82899
*S*_2(2)_	0.52223	*R*_14(2)_	0.36724
*S*_3(2)_	19.5392	*R*_24(2)_	−0.86905

**Table 4:** Summary statistics for population II.

Parameter	Value	Parameter	Value
*N*	50	*S*_2_	0.50508
*n*	15	*S*_3_	21.3175
*λ*	0.04667	*S*_4_	15756
$\bar{X}$	78.2900	*R*_12_	−0.12000
$\bar{R}$	25.5000	*R*_13_	0.22925
*F*(*y*)	0.50000	*R*_23_	−0.78936
*F*(*x*)	0.50000	*R*_14_	0.18435
*S*_1_	0.50508	*R*_24_	−0.86630
Non-response			
Parameter	Value	Parameter	Value
*N*_2_	12	*S*_4(2)_	3.60555
*w*_2_	0.24000	*R*_12(2)_	−0.16903
*λ*_2_	0.01600	*R*_13(2)_	0.25695
*S*_1(2)_	0.51493	*R*_23(2)_	−0.81369
*S*_2(2)_	0.52223	*R*_14(2)_	0.22034
*S*_3(2)_	18.2593	*R*_24(2)_	−0.86905

**Table 5:** Summary statistics for population III.

Parameter	Value	Parameter	Value
*N*	30	*S*_2_	0.50855
*n*	8	*S*_3_	9.23238
*λ*	0.09167	*S*_4_	8.79557
$\bar{X}$	67.2667	*R*_12_	−0.73333
$\bar{R}$	15.5000	*R*_13_	0.71975
*F*(*y*)	0.50000	*R*_23_	−0.83726
*F*(*x*)	0.50000	*R*_14_	0.73622
*S*_1_	0.50855	*R*_24_	−0.86728
Non-response			
Parameter	Value	Parameter	Value
*N*_2_	8	*S*_4(2)_	44949
*w*_2_	0.26667	*R*_12(2)_	−0.60000
*λ*_2_	0.03333	*R*_13(2)_	0.36387
*S*_1(2)_	0.51755	*R*_23(2)_	−0.81227
*S*_2(2)_	0.51755	*R*_14(2)_	0.28172
*S*_3(2)_	9.38749	*R*_24(2)_	−0.84515

**Table 6:** Summary statistics for population IV.

Parameter	Value	Parameter	Value
*N*	69	*S*_2_	0.50361
*n*	20	*S*_3_	7058.99
*λ*	0.03550	$S_{44}^{2}$	20.0602
$\bar{X}$	49544	*R*_12_	0.94202
$\bar{R}$	35.0000	*R*_13_	−0.57090
*F*(*y*)	0.50725	*R*_23_	−0.57274
*F*(*x*)	0.50725	*R*_14_	−0.85584
*S*_1_	0.50361	*R*_24_	−0.86603
Non-response			
Parameter	Value	Parameter	Value
*N*_2_	17	*S*_4(2)_	5.04975
*W*_2_	0.24638	*R*_12(2)_	0.88273
*λ*_2_	0.01231	*R*_13(2)_	−0.62943
*S*_1(2)_	0.51449	*R*_23(2)_	−0.57830
*S*_3(2)_	5920.86	*R*_24(2)_	−0.85391

The PREs of distribution function estimators, computed from five populations, are given in Tables 7–14.

**Table 7:** PREs of distribution function estimators under situation-I using population I.

Estimator	Value	Estimator	Value	Estimator	Value	Estimator	Value	Estimator	Value
${\hat{F}}_{1}(y)$	100	${\hat{F}}_{6}^{\left(1\right)}(y)$	89.193	${\hat{F}}_{7}^{\left(1\right)}(y)$	115.27	${\hat{F}}_{8}^{\left(1\right)}(y)$	116.13	${\hat{F}}_{9}^{\left(1\right)}(y)$	115.36
${\hat{F}}_{2}(y)$	38.87	${\hat{F}}_{6}^{\left(2\right)}(y)$	89.11	${\hat{F}}_{7}^{\left(2\right)}(y)$	115.28	${\hat{F}}_{8}^{\left(2\right)}(y)$	116.13	${\hat{F}}_{9}^{\left(2\right)}(y)$	115.36
${\hat{F}}_{3}(y)$	69.33	${\hat{F}}_{6}^{\left(3\right)}(y)$	89.19	${\hat{F}}_{7}^{\left(3\right)}(y)$	115.27	${\hat{F}}_{8}^{\left(3\right)}(y)$	116.13	${\hat{F}}_{9}^{\left(3\right)}(y)$	115.36
${\hat{F}}_{4}(y)$	108.61	${\hat{F}}_{6}^{\left(4\right)}(y)$	89.03	${\hat{F}}_{7}^{\left(4\right)}(y)$	115.28	${\hat{F}}_{8}^{\left(4\right)}(y)$	116.14	${\hat{F}}_{9}^{\left(4\right)}(y)$	115.37
${\hat{F}}_{5}(y)$	115.07	${\hat{F}}_{6}^{\left(5\right)}(y)$	46.07	${\hat{F}}_{7}^{\left(5\right)}(y)$	121.61	${\hat{F}}_{8}^{\left(5\right)}(y)$	1252	${\hat{F}}_{9}^{\left(5\right)}(y)$	121.70
		${\hat{F}}_{6}^{\left(6\right)}(y)$	46.33	${\hat{F}}_{7}^{\left(6\right)}(y)$	121.5	${\hat{F}}_{8}^{\left(6\right)}(y)$	1241	${\hat{F}}_{9}^{\left(6\right)}(y)$	121.59
		${\hat{F}}_{6}^{\left(7\right)}(y)$	1088	${\hat{F}}_{7}^{\left(7\right)}(y)$	115.09	${\hat{F}}_{8}^{\left(7\right)}(y)$	115.95	${\hat{F}}_{9}^{\left(7\right)}(y)$	115.17
		${\hat{F}}_{6}^{\left(8\right)}(y)$	46.07	${\hat{F}}_{7}^{\left(8\right)}(y)$	121.61	${\hat{F}}_{8}^{\left(8\right)}(y)$	1252	${\hat{F}}_{9}^{\left(8\right)}(y)$	121.70
		${\hat{F}}_{6}^{\left(9\right)}(y)$	1091	${\hat{F}}_{7}^{\left(9\right)}(y)$	115.09	${\hat{F}}_{8}^{\left(9\right)}(y)$	115.95	${\hat{F}}_{9}^{\left(9\right)}(y)$	115.18
		${\hat{F}}_{6}^{\left(10\right)}(y)$	99.44	${\hat{F}}_{7}^{\left(10\right)}(y)$	115.07	${\hat{F}}_{8}^{\left(10\right)}(y)$	115.92	${\hat{F}}_{9}^{\left(10\right)}(y)$	115.15

**Table 8:** PREs of distribution function estimators under situation-II using population I.

Estimator	Value	Estimator	Value	Estimator	Value	Estimator	Value	Estimator	Value
${\hat{F}}_{1}(y)$	100	${\hat{F}}_{6}^{\left(1\right)}(y)$	89.19	${\hat{F}}_{7}^{\left(1\right)}(y)$	115.27	${\hat{F}}_{8}^{\left(1\right)}(y)$	117.91	${\hat{F}}_{9}^{\left(1\right)}(y)$	115.42
${\hat{F}}_{2}(y)$	38.87	${\hat{F}}_{6}^{\left(2\right)}(y)$	89.11	${\hat{F}}_{7}^{\left(2\right)}(y)$	115.28	${\hat{F}}_{8}^{\left(2\right)}(y)$	117.91	${\hat{F}}_{9}^{\left(2\right)}(y)$	115.43
${\hat{F}}_{3}(y)$	69.33	${\hat{F}}_{6}^{\left(3\right)}(y)$	89.19	${\hat{F}}_{7}^{\left(3\right)}(y)$	115.27	${\hat{F}}_{8}^{\left(3\right)}(y)$	117.91	${\hat{F}}_{9}^{\left(3\right)}(y)$	115.42
${\hat{F}}_{4}(y)$	108.61	${\hat{F}}_{6}^{\left(4\right)}(y)$	89.03	${\hat{F}}_{7}^{\left(4\right)}(y)$	115.28	${\hat{F}}_{8}^{\left(4\right)}(y)$	117.92	${\hat{F}}_{9}^{\left(4\right)}(y)$	115.43
${\hat{F}}_{5}(y)$	115.07	${\hat{F}}_{6}^{\left(5\right)}(y)$	46.07	${\hat{F}}_{7}^{\left(5\right)}(y)$	121.61	${\hat{F}}_{8}^{\left(5\right)}(y)$	1241	${\hat{F}}_{9}^{\left(5\right)}(y)$	121.76
		${\hat{F}}_{6}^{\left(6\right)}(y)$	46.33	${\hat{F}}_{7}^{\left(6\right)}(y)$	121.50	${\hat{F}}_{8}^{\left(6\right)}(y)$	1231	${\hat{F}}_{9}^{\left(6\right)}(y)$	121.66
		${\hat{F}}_{6}^{\left(7\right)}(y)$	1088	${\hat{F}}_{7}^{\left(7\right)}(y)$	115.09	${\hat{F}}_{8}^{\left(7\right)}(y)$	117.72	${\hat{F}}_{9}^{\left(7\right)}(y)$	115.24
		${\hat{F}}_{6}^{\left(8\right)}(y)$	46.07	${\hat{F}}_{7}^{\left(8\right)}(y)$	121.61	${\hat{F}}_{8}^{\left(8\right)}(y)$	1241	${\hat{F}}_{9}^{\left(8\right)}(y)$	121.76
		${\hat{F}}_{6}^{\left(9\right)}(y)$	1091	${\hat{F}}_{7}^{\left(9\right)}(y)$	115.09	${\hat{F}}_{8}^{\left(9\right)}(y)$	117.72	${\hat{F}}_{9}^{\left(9\right)}(y)$	115.24
		${\hat{F}}_{6}^{\left(10\right)}(y)$	99.44	${\hat{F}}_{7}^{\left(10\right)}(y)$	115.07	${\hat{F}}_{8}^{\left(10\right)}(y)$	117.70	${\hat{F}}_{9}^{\left(10\right)}(y)$	115.22

**Table 9:** PREs of distribution function estimators under situation-I using population II.

Estimator	Value	Estimator	Value	Estimator	Value	Estimator	Value	Estimator	Value
${\hat{F}}_{1}(y)$	100	${\hat{F}}_{6}^{\left(1\right)}(y)$	93.54	${\hat{F}}_{7}^{\left(1\right)}(y)$	109.64	${\hat{F}}_{8}^{\left(1\right)}(y)$	1112	${\hat{F}}_{9}^{\left(1\right)}(y)$	111.94
${\hat{F}}_{2}(y)$	49.21	${\hat{F}}_{6}^{\left(2\right)}(y)$	93.49	${\hat{F}}_{7}^{\left(2\right)}(y)$	109.64	${\hat{F}}_{8}^{\left(2\right)}(y)$	1112	${\hat{F}}_{9}^{\left(2\right)}(y)$	111.94
${\hat{F}}_{3}(y)$	69.33	${\hat{F}}_{6}^{\left(3\right)}(y)$	93.54	${\hat{F}}_{7}^{\left(3\right)}(y)$	109.64	${\hat{F}}_{8}^{\left(3\right)}(y)$	1112	${\hat{F}}_{9}^{\left(3\right)}(y)$	111.94
${\hat{F}}_{4}(y)$	103.04	${\hat{F}}_{6}^{\left(4\right)}(y)$	93.44	${\hat{F}}_{7}^{\left(4\right)}(y)$	109.65	${\hat{F}}_{8}^{\left(4\right)}(y)$	1112	${\hat{F}}_{9}^{\left(4\right)}(y)$	111.94
${\hat{F}}_{5}(y)$	109.5	${\hat{F}}_{6}^{\left(5\right)}(y)$	56.89	${\hat{F}}_{7}^{\left(5\right)}(y)$	113.76	${\hat{F}}_{8}^{\left(5\right)}(y)$	118.43	${\hat{F}}_{9}^{\left(5\right)}(y)$	116.15
		${\hat{F}}_{6}^{\left(6\right)}(y)$	57.17	${\hat{F}}_{7}^{\left(6\right)}(y)$	113.69	${\hat{F}}_{8}^{\left(6\right)}(y)$	118.36	${\hat{F}}_{9}^{\left(6\right)}(y)$	116.08
		${\hat{F}}_{6}^{\left(7\right)}(y)$	101.42	${\hat{F}}_{7}^{\left(7\right)}(y)$	109.51	${\hat{F}}_{8}^{\left(7\right)}(y)$	113.99	${\hat{F}}_{9}^{\left(7\right)}(y)$	111.81
		${\hat{F}}_{6}^{\left(8\right)}(y)$	56.89	${\hat{F}}_{7}^{\left(8\right)}(y)$	113.76	${\hat{F}}_{8}^{\left(8\right)}(y)$	118.43	${\hat{F}}_{9}^{\left(8\right)}(y)$	116.15
		${\hat{F}}_{6}^{\left(9\right)}(y)$	101.43	${\hat{F}}_{7}^{\left(9\right)}(y)$	109.51	${\hat{F}}_{8}^{\left(9\right)}(y)$	113.99	${\hat{F}}_{9}^{\left(9\right)}(y)$	111.81
		${\hat{F}}_{6}^{\left(10\right)}(y)$	99.70	${\hat{F}}_{7}^{\left(10\right)}(y)$	109.50	${\hat{F}}_{8}^{\left(10\right)}(y)$	113.97	${\hat{F}}_{9}^{\left(10\right)}(y)$	111.79

**Table 10:** PREs of distribution function estimators under situation-II using population II.

Estimator	Value	Estimator	Value	Estimator	Value	Estimator	Value	Estimator	Value
${\hat{F}}_{1}(y)$	100	${\hat{F}}_{6}^{\left(1\right)}(y)$	95.28	${\hat{F}}_{7}^{\left(1\right)}(y)$	107.67	${\hat{F}}_{8}^{\left(1\right)}(y)$	111.43	${\hat{F}}_{9}^{\left(1\right)}(y)$	109.67
${\hat{F}}_{2}(y)$	524	${\hat{F}}_{6}^{\left(2\right)}(y)$	95.24	${\hat{F}}_{7}^{\left(2\right)}(y)$	107.67	${\hat{F}}_{8}^{\left(2\right)}(y)$	111.43	${\hat{F}}_{9}^{\left(2\right)}(y)$	109.68
${\hat{F}}_{3}(y)$	609	${\hat{F}}_{6}^{\left(3\right)}(y)$	95.28	${\hat{F}}_{7}^{\left(3\right)}(y)$	107.67	${\hat{F}}_{8}^{\left(3\right)}(y)$	111.43	${\hat{F}}_{9}^{\left(3\right)}(y)$	109.67
${\hat{F}}_{4}(y)$	101.07	${\hat{F}}_{6}^{\left(4\right)}(y)$	95.20	${\hat{F}}_{7}^{\left(4\right)}(y)$	107.68	${\hat{F}}_{8}^{\left(4\right)}(y)$	111.43	${\hat{F}}_{9}^{\left(4\right)}(y)$	109.67
${\hat{F}}_{5}(y)$	107.53	${\hat{F}}_{6}^{\left(5\right)}(y)$	69.66	${\hat{F}}_{7}^{\left(5\right)}(y)$	109.98	${\hat{F}}_{8}^{\left(5\right)}(y)$	113.82	${\hat{F}}_{9}^{\left(5\right)}(y)$	1102
		${\hat{F}}_{6}^{\left(6\right)}(y)$	69.78	${\hat{F}}_{7}^{\left(6\right)}(y)$	109.96	${\hat{F}}_{8}^{\left(6\right)}(y)$	113.81	${\hat{F}}_{9}^{\left(6\right)}(y)$	1100
		${\hat{F}}_{6}^{\left(7\right)}(y)$	100.49	${\hat{F}}_{7}^{\left(7\right)}(y)$	107.54	${\hat{F}}_{8}^{\left(7\right)}(y)$	111.29	${\hat{F}}_{9}^{\left(7\right)}(y)$	109.53
		${\hat{F}}_{6}^{\left(8\right)}(y)$	69.66	${\hat{F}}_{7}^{\left(8\right)}(y)$	109.98	${\hat{F}}_{8}^{\left(8\right)}(y)$	113.82	${\hat{F}}_{9}^{\left(8\right)}(y)$	1102
		${\hat{F}}_{6}^{\left(9\right)}(y)$	100.49	${\hat{F}}_{7}^{\left(9\right)}(y)$	107.54	${\hat{F}}_{8}^{\left(9\right)}(y)$	111.29	${\hat{F}}_{9}^{\left(9\right)}(y)$	109.53
		${\hat{F}}_{6}^{\left(10\right)}(y)$	99.82	${\hat{F}}_{7}^{\left(10\right)}(y)$	107.53	${\hat{F}}_{8}^{\left(10\right)}(y)$	111.28	${\hat{F}}_{9}^{\left(10\right)}(y)$	109.52

**Table 11:** PREs of distribution function estimators under situation-I using population III.

Estimator	Value	Estimator	Value	Estimator	Value	Estimator	Value	Estimator	Value
${\hat{F}}_{1}(y)$	100	${\hat{F}}_{6}^{\left(1\right)}(y)$	79.57	${\hat{F}}_{7}^{\left(1\right)}(y)$	208.21	${\hat{F}}_{8}^{\left(1\right)}(y)$	210.57	${\hat{F}}_{9}^{\left(1\right)}(y)$	216.06
${\hat{F}}_{2}(y)$	29.47	${\hat{F}}_{6}^{\left(2\right)}(y)$	79.36	${\hat{F}}_{7}^{\left(2\right)}(y)$	208.22	${\hat{F}}_{8}^{\left(2\right)}(y)$	210.59	${\hat{F}}_{9}^{\left(2\right)}(y)$	216.07
${\hat{F}}_{3}(y)$	1694	${\hat{F}}_{6}^{\left(3\right)}(y)$	79.57	${\hat{F}}_{7}^{\left(3\right)}(y)$	208.21	${\hat{F}}_{8}^{\left(3\right)}(y)$	210.57	${\hat{F}}_{9}^{\left(3\right)}(y)$	216.06
${\hat{F}}_{4}(y)$	1940	${\hat{F}}_{6}^{\left(4\right)}(y)$	79.16	${\hat{F}}_{7}^{\left(4\right)}(y)$	208.24	${\hat{F}}_{8}^{\left(4\right)}(y)$	210.61	${\hat{F}}_{9}^{\left(4\right)}(y)$	216.09
${\hat{F}}_{5}(y)$	207.46	${\hat{F}}_{6}^{\left(5\right)}(y)$	152.74	${\hat{F}}_{7}^{\left(5\right)}(y)$	270.64	${\hat{F}}_{8}^{\left(5\right)}(y)$	2709	${\hat{F}}_{9}^{\left(5\right)}(y)$	282.08
		${\hat{F}}_{6}^{\left(6\right)}(y)$	1424	${\hat{F}}_{7}^{\left(6\right)}(y)$	2855	${\hat{F}}_{8}^{\left(6\right)}(y)$	288.28	${\hat{F}}_{9}^{\left(6\right)}(y)$	296.98
		${\hat{F}}_{6}^{\left(7\right)}(y)$	145.64	${\hat{F}}_{7}^{\left(7\right)}(y)$	209.72	${\hat{F}}_{8}^{\left(7\right)}(y)$	2110	${\hat{F}}_{9}^{\left(7\right)}(y)$	217.63
		${\hat{F}}_{6}^{\left(8\right)}(y)$	152.74	${\hat{F}}_{7}^{\left(8\right)}(y)$	270.64	${\hat{F}}_{8}^{\left(8\right)}(y)$	2709	${\hat{F}}_{9}^{\left(8\right)}(y)$	282.08
		${\hat{F}}_{6}^{\left(9\right)}(y)$	146.98	${\hat{F}}_{7}^{\left(9\right)}(y)$	209.85	${\hat{F}}_{8}^{\left(9\right)}(y)$	2124	${\hat{F}}_{9}^{\left(9\right)}(y)$	217.76
		${\hat{F}}_{6}^{\left(10\right)}(y)$	97.78	${\hat{F}}_{7}^{\left(10\right)}(y)$	207.47	${\hat{F}}_{8}^{\left(10\right)}(y)$	209.83	${\hat{F}}_{9}^{\left(10\right)}(y)$	215.29

**Table 12:** PREs of distribution function estimators under situation-II using population III.

Estimator	Value	Estimator	Value	Estimator	Value	Estimator	Value	Estimator	Value
${\hat{F}}_{1}(y)$	100	${\hat{F}}_{6}^{\left(1\right)}(y)$	83.66	${\hat{F}}_{7}^{\left(1\right)}(y)$	177.62	${\hat{F}}_{8}^{\left(1\right)}(y)$	185.30	${\hat{F}}_{9}^{\left(1\right)}(y)$	185.98
${\hat{F}}_{2}(y)$	35.82	${\hat{F}}_{6}^{\left(2\right)}(y)$	83.49	${\hat{F}}_{7}^{\left(2\right)}(y)$	177.63	${\hat{F}}_{8}^{\left(2\right)}(y)$	185.32	${\hat{F}}_{9}^{\left(2\right)}(y)$	185.99
${\hat{F}}_{3}(y)$	151.28	${\hat{F}}_{6}^{\left(3\right)}(y)$	83.66	${\hat{F}}_{7}^{\left(3\right)}(y)$	177.62	${\hat{F}}_{8}^{\left(3\right)}(y)$	185.30	${\hat{F}}_{9}^{\left(3\right)}(y)$	185.98
${\hat{F}}_{4}(y)$	1611	${\hat{F}}_{6}^{\left(4\right)}(y)$	83.32	${\hat{F}}_{7}^{\left(4\right)}(y)$	177.65	${\hat{F}}_{8}^{\left(4\right)}(y)$	185.33	${\hat{F}}_{9}^{\left(4\right)}(y)$	186.00
${\hat{F}}_{5}(y)$	177.16	${\hat{F}}_{6}^{\left(5\right)}(y)$	1443	${\hat{F}}_{7}^{\left(5\right)}(y)$	207.48	${\hat{F}}_{8}^{\left(5\right)}(y)$	216.89	${\hat{F}}_{9}^{\left(5\right)}(y)$	217.71
		${\hat{F}}_{6}^{\left(6\right)}(y)$	138.06	${\hat{F}}_{7}^{\left(6\right)}(y)$	2184	${\hat{F}}_{8}^{\left(6\right)}(y)$	2262	${\hat{F}}_{9}^{\left(6\right)}(y)$	223.48
		${\hat{F}}_{6}^{\left(7\right)}(y)$	132.03	${\hat{F}}_{7}^{\left(7\right)}(y)$	178.54	${\hat{F}}_{8}^{\left(7\right)}(y)$	186.26	${\hat{F}}_{9}^{\left(7\right)}(y)$	186.94
		${\hat{F}}_{6}^{\left(8\right)}(y)$	1443	${\hat{F}}_{7}^{\left(8\right)}(y)$	207.48	${\hat{F}}_{8}^{\left(8\right)}(y)$	216.89	${\hat{F}}_{9}^{\left(8\right)}(y)$	217.71
		${\hat{F}}_{6}^{\left(9\right)}(y)$	132.90	${\hat{F}}_{7}^{\left(9\right)}(y)$	178.62	${\hat{F}}_{8}^{\left(9\right)}(y)$	186.34	${\hat{F}}_{9}^{\left(9\right)}(y)$	187.02
		${\hat{F}}_{6}^{\left(10\right)}(y)$	98.29	${\hat{F}}_{7}^{\left(10\right)}(y)$	177.17	${\hat{F}}_{8}^{\left(10\right)}(y)$	1883	${\hat{F}}_{9}^{\left(10\right)}(y)$	185.50

**Table 13:** PREs of distribution function estimators under situation-I using population IV.

Estimator	Value	Estimator	Value	Estimator	Value	Estimator	Value	Estimator	Value
${\hat{F}}_{1}(y)$	100	${\hat{F}}_{6}^{\left(1\right)}(y)$	140.20	${\hat{F}}_{7}^{\left(1\right)}(y)$	715.05	${\hat{F}}_{8}^{\left(1\right)}(y)$	736.10	${\hat{F}}_{9}^{\left(1\right)}(y)$	755.46
${\hat{F}}_{2}(y)$	678.05	${\hat{F}}_{6}^{\left(2\right)}(y)$	139.93	${\hat{F}}_{7}^{\left(2\right)}(y)$	715.03	${\hat{F}}_{8}^{\left(2\right)}(y)$	736.09	${\hat{F}}_{9}^{\left(2\right)}(y)$	755.44
${\hat{F}}_{3}(y)$	25.76	${\hat{F}}_{6}^{\left(3\right)}(y)$	140.23	${\hat{F}}_{7}^{\left(3\right)}(y)$	715.05	${\hat{F}}_{8}^{\left(3\right)}(y)$	736.10	${\hat{F}}_{9}^{\left(3\right)}(y)$	755.46
${\hat{F}}_{4}(y)$	709.37	${\hat{F}}_{6}^{\left(4\right)}(y)$	139.69	${\hat{F}}_{7}^{\left(4\right)}(y)$	715.02	${\hat{F}}_{8}^{\left(4\right)}(y)$	736.08	${\hat{F}}_{9}^{\left(4\right)}(y)$	755.43
${\hat{F}}_{5}(y)$	7103	${\hat{F}}_{6}^{\left(5\right)}(y)$	141.99	${\hat{F}}_{7}^{\left(5\right)}(y)$	715.12	${\hat{F}}_{8}^{\left(5\right)}(y)$	736.18	${\hat{F}}_{9}^{\left(5\right)}(y)$	755.54
		${\hat{F}}_{6}^{\left(6\right)}(y)$	141.75	${\hat{F}}_{7}^{\left(6\right)}(y)$	715.11	${\hat{F}}_{8}^{\left(6\right)}(y)$	736.17	${\hat{F}}_{9}^{\left(6\right)}(y)$	755.53
		${\hat{F}}_{6}^{\left(7\right)}(y)$	138.24	${\hat{F}}_{7}^{\left(7\right)}(y)$	7196	${\hat{F}}_{8}^{\left(7\right)}(y)$	736.02	${\hat{F}}_{9}^{\left(7\right)}(y)$	755.37
		${\hat{F}}_{6}^{\left(8\right)}(y)$	1402	${\hat{F}}_{7}^{\left(8\right)}(y)$	715.12	${\hat{F}}_{8}^{\left(8\right)}(y)$	736.18	${\hat{F}}_{9}^{\left(8\right)}(y)$	755.54
		${\hat{F}}_{6}^{\left(9\right)}(y)$	137.98	${\hat{F}}_{7}^{\left(9\right)}(y)$	7195	${\hat{F}}_{8}^{\left(9\right)}(y)$	736.00	${\hat{F}}_{9}^{\left(9\right)}(y)$	755.36
		${\hat{F}}_{6}^{\left(10\right)}(y)$	101.35	${\hat{F}}_{7}^{\left(10\right)}(y)$	7103	${\hat{F}}_{8}^{\left(10\right)}(y)$	735.06	${\hat{F}}_{9}^{\left(10\right)}(y)$	7538

**Table 14:** PREs of distribution function estimators under situation-II using population IV.

Estimator	Value	Estimator	Value	Estimator	Value	Estimator	Value	Estimator	Value
${\hat{F}}_{1}(y)$	100	${\hat{F}}_{6}^{\left(1\right)}(y)$	127.83	${\hat{F}}_{7}^{\left(1\right)}(y)$	3060	${\hat{F}}_{8}^{\left(1\right)}(y)$	305.59	${\hat{F}}_{9}^{\left(1\right)}(y)$	308.98
${\hat{F}}_{2}(y)$	297.38	${\hat{F}}_{6}^{\left(2\right)}(y)$	127.66	${\hat{F}}_{7}^{\left(2\right)}(y)$	3059	${\hat{F}}_{8}^{\left(2\right)}(y)$	305.58	${\hat{F}}_{9}^{\left(2\right)}(y)$	308.98
${\hat{F}}_{3}(y)$	31.59	${\hat{F}}_{6}^{\left(3\right)}(y)$	127.85	${\hat{F}}_{7}^{\left(3\right)}(y)$	3059	${\hat{F}}_{8}^{\left(3\right)}(y)$	305.59	${\hat{F}}_{9}^{\left(3\right)}(y)$	308.98
${\hat{F}}_{4}(y)$	299.62	${\hat{F}}_{6}^{\left(4\right)}(y)$	127.50	${\hat{F}}_{7}^{\left(4\right)}(y)$	3059	${\hat{F}}_{8}^{\left(4\right)}(y)$	305.58	${\hat{F}}_{9}^{\left(4\right)}(y)$	308.98
${\hat{F}}_{5}(y)$	3029	${\hat{F}}_{6}^{\left(5\right)}(y)$	128.96	${\hat{F}}_{7}^{\left(5\right)}(y)$	3062	${\hat{F}}_{8}^{\left(5\right)}(y)$	305.60	${\hat{F}}_{9}^{\left(5\right)}(y)$	309.00
		${\hat{F}}_{6}^{\left(6\right)}(y)$	128.80	${\hat{F}}_{7}^{\left(6\right)}(y)$	3062	${\hat{F}}_{8}^{\left(6\right)}(y)$	305.60	${\hat{F}}_{9}^{\left(6\right)}(y)$	309.00
		${\hat{F}}_{6}^{\left(7\right)}(y)$	126.58	${\hat{F}}_{7}^{\left(7\right)}(y)$	3057	${\hat{F}}_{8}^{\left(7\right)}(y)$	305.56	${\hat{F}}_{9}^{\left(7\right)}(y)$	308.96
		${\hat{F}}_{6}^{\left(8\right)}(y)$	128.98	${\hat{F}}_{7}^{\left(8\right)}(y)$	3062	${\hat{F}}_{8}^{\left(8\right)}(y)$	305.61	${\hat{F}}_{9}^{\left(8\right)}(y)$	309.00
		${\hat{F}}_{6}^{\left(9\right)}(y)$	126.41	${\hat{F}}_{7}^{\left(9\right)}(y)$	3057	${\hat{F}}_{8}^{\left(9\right)}(y)$	305.56	${\hat{F}}_{9}^{\left(9\right)}(y)$	308.96
		${\hat{F}}_{6}^{\left(10\right)}(y)$	101.02	${\hat{F}}_{7}^{\left(10\right)}(y)$	3029	${\hat{F}}_{8}^{\left(10\right)}(y)$	305.28	${\hat{F}}_{9}^{\left(10\right)}(y)$	308.67

**Population I** -Gujarati [34]
*Y*: The eggs produced in 1990 (millions) and
*X*: The price per dozen (cents) in 1991. The proportion of the non-response units in the given population is considered to be the last 25% units.
**Population II** -Gujarati [34]
*Y*: The eggs produced in 1990 (millions) and
*X*: The price per dozen (cents) in 1990. The proportion of the non-response units in the given population is considered to be the last 25% units.
**Population III**-Singh [35]
*Y*: Duration of sleep of persons with age more than 50 years and
*X*: The age of persons in years. The proportion of the non-response units in the given population is considered to be the last 25% units.
**Population IV**-Singh [35]
*Y*: The estimated number of fish caught by marine recreational fisherman in year 1995 and
*X*: The estimated number of fish caught by marine recreational fisherman in year 199 The share of the non-response units in the given population is the last 25% units.

As mention above, we used four data sets for numerical illustration. We also considered different sample sizes from these populations. We then used simple random sampling, and the MSEs (minimum) of the proposed families of estimators pointed out in Eqs 28 and 21. The proposed families of estimators and the adapted estimators were compared between each other with respect to their PREs values. The result of PREs are presented in Tables 7–14, for both Situations under non-response. In Tables 3–6, we see the summary statistics about the populations. We can also observed from numerical results shown in Tables 7–14 that the PREs of all families of estimators change with the choices of a and b. It is further noted that the proposed families of estimators are more precise than the adapted distribution function estimators of Hansen and Hurwitz [1], Cochran [28], Murthy [29], Rao [30], Singh et al. [31] and Grover and Kaur [32] in terms of the PRE. It can be seen that, for some data sets the second proposed family of the estimators perform better than the first proposed family of estimators for both situations of non-response, and for data sets, it is also observed that the first proposed family of the estimators performs better than the second proposed family of estimators under both situations of non-response.

## 7. Conclusion

In this paper, we have proposed two new families of estimators for estimating the finite population distribution function in the presence of non-response under simple random sampling. The proposed estimators required information on the sample distribution function of study variable, and the distribution function of auxiliary variable with additional information on either sample mean or ranks of the auxiliary variable. The bias and MSE of the proposed families of estimators were derived under the first order of approximation. Based on the theoretical and numerical (four populations considered in the numerical investigation) comparisons, it has turned out that the proposed families of estimators are more precise than their adaptive estimators under two situations of non-response. Thus, we acclaim categorically, the use of our proposed families of estimators over the existing estimators deliberate in this paper for the new survey for estimating the finite population distribution function in the presence of non-response.

## References

[1] Hansen MH, Hurwitz WN. The problem of non-response in sample surveys. Journal of the American Statistical Association. 1946 12 1;41(236):517–529. 10.1080/01621459.1946.10501894 20279350

[2] Rao PS. Ratio estimation with sub sampling the non-respondents. Survey methodology. 1986 12;12(2):217–230.

[3] Khare BB, Srivastava S. Transformed ratio type estimators for the population mean in the presence of nonresponse. Communications in Statistics-Theory and Methods. 1997 1 1;26(7):1779–1791.

[4] Khare BB, Sinha RR. On class of estimators for population mean using multi-auxiliary characters in the presence of non-response. Statistics in Transition. 2009;10(1):3–14.

[5] Khare BB, Sinha RR. Estimation of population mean using multi-auxiliary characters with subsampling the nonrespondents. Statistics in Transition new aeries. 2011;1(12):45–56.

[6] Yunusa O, Kumar S. Ratio-cum-product estimator using exponential estimator in the presence of non-response. Journal of Advanced Computing. 2014;3(1):1–11.

[7] Muneer S, Shabbir J, Khalil A. Estimation of finite population mean in simple random sampling and stratified random sampling using two auxiliary variables. Communications in Statistics-Theory and Methods. 2017 3 4;46(5):2181–2192.

[8] Pal SK, Singh HP. Estimation of finite population mean using auxiliary information in presence of non-response. Communications in Statistics-Simulation and Computation. 2018 1 2;47(1):143–165.

[9] Ahmad S, Shabbir J. Use of extreme values to estimate finite population mean under pps sampling scheme. Journal of Reliability and Statistical Studies. 2018 10 5:99–112.

[10] Chambers RL, Dunstan R. Estimating distribution functions from survey data. Biometrika. 1986 12 1;73(3):597–604.

[11] Rao JN, Kovar JG, Mantel HJ. On estimating distribution functions and quantiles from survey data using auxiliary information. Biometrika. 1990 6 1:365–375.

[12] Rao TJ. Estimating totals and distribution functions using auxiliary information at the estimation stage. Journal of official statistics. 1994; 10(2):153.

[13] Kuk AY. A kernel method for estimating finite population distribution functions using auxiliary information. Biometrika. 1993 6 1;80(2):385–392.

[14] Ahmed MS. and Abu-Dayyeh W. Estimation of finite-population distribution function using multivariate auxiliary information. Statistics in Transition. 2001 5(3):501–507

[15] Chen J, Wu C. Estimation of distribution function and quantiles using the model-calibrated pseudo empirical likelihood method. Statistica Sinica. 2002 10 1:1223–1239.

[16] Rueda M, Martínez S, Martínez H, Arcos A. Estimation of the distribution function with calibration methods. Journal of statistical planning and inference. 2007 2 1;137(2):435–448.

[17] Singh HP, Singh S, Kozak M. A family of estimators of finite-population distribution function using auxiliary information. Acta applicandae mathematicae. 2008 11 1;104(2):115–130.

[18] Chen F, Chen S, Ma X. Crash frequency modeling using real-time environmental and traffic data and unbalanced panel data models. International journal of environmental research and public health. 2016 6;13(6):609 10.3390/ijerph13060609 27322306 PMC4924066

[19] Zeng Q, Wen H, Huang H, Pei X, Wong SC. A multivariate random-parameters Tobit model for analyzing highway crash rates by injury severity. Accident Analysis & Prevention. 2017 2 1;99:184–191. 10.1016/j.aap.2016.11.018 27914307

[20] Yaqub M, Shabbir J. Estimation of population distribution function in the presence of non-response. Hacettepe Journal of Mathematics and Statistics. 2018 4 1;47(2):471–511.

[21] Dong B, Ma X, Chen F, Chen S. Investigating the differences of single-vehicle and multivehicle accident probability using mixed logit model. Journal of Advanced Transportation. 2018 1 1;2018 10.1155/2018/7905140 32908973 PMC7477825

[22] Chen F, Chen S, Ma X. Analysis of hourly crash likelihood using unbalanced panel data mixed logit model and real-time driving environmental big data. Journal of safety research. 2018 6 1;65:153–159. 10.1016/j.jsr.2018.02.010 29776524

[23] Zeng Q, Guo Q, Wong SC, Wen H, Huang H, Pei X,. Jointly modeling area-level crash rates by severity: a bayesian multivariate random-parameters spatio-temporal tobit regression. Transportmetrica A: Transport science. 2019 8 21; 15(2):1867–188.

[24] Zeng Q, Wen H, Wong SC, Huang H, Guo Q, Pei X. Spatial joint analysis for zonal day time and night time crash frequencies using a Bayesian bivariate conditional autoregressive model. Journal of Transportation Safety & Security. 2020 4 20;12(4):566–585. 10.4271/2016-01-1439 27648455 PMC5026383

[25] Hussain S, Zichuan M, Hussain S, Iftikhar A, Asif M, Akhtar S, et al On Estimation of Distribution Function Using Dual Auxiliary Information under Nonresponse Using Simple Random Sampling. Journal of Probability and Statistics. 2020 9 26;2020.

[26] Hussain S, Ahmad S, Saleem M, Akhtar S. Finite population distribution function estimation with dual use of auxiliary information under simple and stratified random sampling. Plos one. 2020 9 28;15(9):e0239098 10.1371/journal.pone.0239098 32986764 PMC7521711

[27] Plewes TJ, Tourangeau R, editors. Nonresponse in social science surveys: A research agenda. National Academies Press; 2013.

[28] Cochran WG. The estimation of the yields of cereal experiments by sampling for the ratio of grain to total produce. The journal of agricultural science. 1940 4;30(2):262–275.

[29] Murthy MN. Product method of estimation. Sankhyā: The Indian Journal of Statistics, Series A. 1964 7 1; 26(1):69–74.

[30] Rao TJ. On certail methods of improving ration and regression estimators. Communications in Statistics-Theory and Methods. 1991 1 1;20(10):3325–3340.

[31] Singh R, Chauhan P, Sawan N, Smarandache F. Improvement in estimating the population mean using exponential estimator in simple random sampling. Auxiliary Information and a priori Values in Construction of Improved Estimators. 2007:33.

[32] Grover LK, Kaur P. A generalized class of ratio type exponential estimators of population mean under linear transformation of auxiliary variable. Communications in Statistics-Simulation and Computation. 2014 1 1;43(7):1552–1574.

[33] Haq A, Khan M, Hussain Z. A new estimator of finite population mean based on the dual use of the auxiliary information. Communications in Statistics-Theory and Methods. 2017 5 3;46(9):4425–4436.

[34] Gujarati DN. Basic econometrics. Tata McGraw-Hill Education; 2009.

[35] Singh S. (2003). Advanced Sampling Theory with Applications: How Michael ‘Selected’ Amy, Volume 1. Springer Science & Business Media.

